# Effects on Maternal Mental Health and Parental Functioning of an Interdisciplinary Intervention to Support Women in Vulnerable Positions Through Pregnancy and Early Motherhood: A Randomized Controlled Trial

**DOI:** 10.3390/healthcare13131505

**Published:** 2025-06-24

**Authors:** Lene Nygaard, Jonas Cuzulan Hirani, Mette Friis-Hansen, Deborah Davis, Ellen Aagaard Nøhr, Maiken Pontoppidan

**Affiliations:** 1 Research Unit for Gynecology and Obstetrics, Department of Clinical Research, University of Southern Denmark, 5230 Odense, Denmark; eanohr@health.sdu.dk; 2 Department of Gynecology and Obstetrics, Odense University Hospital, 5000 Odense, Denmark; 3 VIVE–The Danish Centre for Social Science Research, 1052 Copenhagen, Denmark; jjh@vive.dk (J.C.H.); mfh@vive.dk (M.F.-H.); mpo@vive.dk (M.P.); 4 Faculty of Health, University of Canberra and ACT Health, Bruce, ACT 2617, Australia; deborah.davis@canberra.edu.au

**Keywords:** randomized controlled trial, early intervention, intersectoral collaboration, pregnancy, parents, mental health

## Abstract

**Background/Objectives:** The transition to motherhood can be particularly challenging for women with limited socioeconomic resources or mental health concerns. The FAmily Clinic And Municipality (FACAM) intervention was designed to provide additional support through health visitors or family therapists, starting in pregnancy and continuing until the child reached school age. This paper evaluates the effects of the FACAM intervention on the secondary outcomes, maternal mental health and parental functioning during the child’s first year of life. **Methods:** A total of 331 pregnant women were randomized to either the FACAM intervention (n = 163) or usual care (n = 168). Participants completed questionnaires at baseline and at 3 (N = 284) and 12 (N = 248) months postpartum. Outcomes included maternal mental well-being, satisfaction with motherhood, depressive symptoms, parental stress, parental reflective functioning, worries, and breastfeeding duration. **Results:** At 12 months postpartum, FACAM mothers reported greater concern about housing issues (b = 0.56, 95% CI [0.06, 1.06], *p* = 0.03). No other significant differences in the reported outcomes were observed between the groups. **Conclusion:** The FACAM intervention did not demonstrate superiority over usual care in improving maternal mental health and parental functioning during the first year postpartum. The high-quality and needs-based approach of standard care in Denmark may have limited the potential for additional interventions to yield measurable improvements in maternal outcomes.

## 1. Introduction

Pregnancy and the transition to motherhood bring profound psychological, physiological, and social changes [1]. These adjustments can be particularly challenging for women facing mental health issues or limited social and financial resources. The critical bond between mother and child begins during pregnancy but may be disrupted if the mother is burdened by emotional or social difficulties or mental health conditions [2,3,4].

In high-income countries, perinatal mental health challenges are common. Depression affects approximately 11% of women during pregnancy or the early postpartum period [5]. Danish registry data from 2000 to 2022 report incidence rates of 34.3 per 10,000 person-years for hospital-diagnosed postpartum depression and 18.9 for general depression among women aged 15–49 [6]. Anxiety symptoms are similarly prevalent, with rates in high-income countries of 19.4 during pregnancy and 13.7 in the first six months postpartum [7], and up to 21% of women experience an anxiety disorder during pregnancy or within the first 12 months after birth [8]. Mental health challenges during pregnancy, such as depression and anxiety disorders, are linked to adverse pregnancy outcomes, including preterm birth and low birth weight [9].

Despite the high prevalence of perinatal mental health problems, many women face substantial barriers to seeking help. These include limited awareness of the severity of problems, sociocultural factors such as language barriers, and cultural values about mental illness. Fragmentation in care systems and lack of continuity further hinder access and engagement, particularly for women of minority ethnic backgrounds [10].

In addition, a history of childhood trauma, including neglect or abuse, can result in poorer physical and mental health in adulthood [11] and increases the risk of intergenerational transmission of neglect and maltreatment [12,13,14]. These risks are further compounded by socioeconomic disadvantage. Women with fewer financial and social resources are more likely to experience adverse perinatal outcomes, including complications during pregnancy, lower birth weight, and poorer maternal health [15]. Low health literacy, especially among socioeconomically deprived or ethnic minority groups, has also been linked to suboptimal perinatal outcomes [16].

Finally, children whose mothers suffer from severe mental illness are at increased risk of developing pediatric or mental disorders during early childhood [17]. These factors underscore the importance of early, accessible, and targeted interventions to support parental mental health and strengthen the parent–child relationship.

Systematic reviews have documented the effects of early parenting interventions, but these vary substantially in terms of target populations (universal vs. at-risk; pregnancy vs. postnatal; infants vs. toddlers), intervention types (group-based, dyadic, attachment-based, psychodynamic, eHealth), and outcomes assessed (child development, parent–child relationship, maternal mental health) [18,19,20,21,22,23,24,25,26]. For example, Barlow et al. (2016) [18] focused on group-based parenting programs for improving child behavior in universal and at-risk populations with children under four, while Rayce et al. (2017) [19] reviewed structured interventions for at-risk parents with infants aged 0–12 months. Reviews also differ in timing and delivery context—some target pregnancy [22,27], others the postnatal period [23,24]—as well as in their underlying mechanisms of change, such as enhancing sensitivity, attachment security, or reflective functioning.

Although results are mixed, several intervention types have shown promising effects: group- and individual-based programs report short-term improvements in child behavior and parental sensitivity [18,19]; interventions delivered in primary care may benefit child socioemotional outcomes [20]; and programs targeting reflective functioning or attachment show reductions in disorganized attachment and, in some cases, improved parental mentalization [23,24]. Moreover, interventions initiated during pregnancy or early infancy appear to reduce maternal depressive symptoms [22]. Still, effect sizes and outcome domains differ, and many reviews stress the need for more rigorous, long-term evaluations—particularly for attachment and relational outcomes [22,24,25].

Despite this growing international evidence base, comparable interventions have not been widely implemented or evaluated in Denmark—particularly those initiated during pregnancy. There remains a significant need for interventions that address the interconnected challenges faced by families in vulnerable positions, such as mental and physical health problems, parenting challenges, and social adversity. These interventions should also build on the documented benefits of continuity of care, which has been associated with improved maternal outcomes and birth experiences [28].

The FAmily Clinic And Municipality (FACAM) intervention was developed to address the need for comprehensive early parenting support tailored to pregnant women in vulnerable situations. The intervention included contact with a support person (referred to as the FACAM person) and an invitation to participate in either individual sessions or an attachment-based parenting course. The intervention was theoretically grounded in mentalization, a concept referring to the ability to understand one’s own and others’ mental states [29], which has shown relevance for improving parental functioning and the parent–child relationship [30,31]. We conducted a randomized controlled trial to examine the effects of FACAM on a range of outcomes, including the mother–child relationship, maternal social functioning, mental health, reflective functioning, well-being, parental stress, and the development and well-being of the child. While the primary outcome, focusing on maternal sensitivity and parent–child interaction, has been reported elsewhere [32], this paper focuses on secondary outcomes. Specifically, it examines the effects of the FACAM intervention on outcomes related to maternal mental health, well-being, and parental functioning at 3 and 12 months postpartum.

## 2. Materials and Methods

The study is a prospective, superiority, parallel, 1:1 randomized controlled trial (RCT). We randomized 331 pregnant women to receive either the FACAM intervention or care as usual (CAU). The trial follows the guidelines outlined in the Consolidated Standards of Reporting Trials (CONSORT) statement. The Committee on Health Research in the Region of Southern Denmark approved the study (Journal No. 18/48509). The internal review board of VIVE (The Danish Center for Social Science Research) provided ethical approval. Additionally, the Committee on Health Research Ethics in the Region of Southern Denmark reviewed the study protocol and concluded that no further ethical approval was necessary (Case No. S-20182000-110).

### 2.1. Participants and Recruitment

The trial recruited pregnant women categorized into care group 3 or 4, as defined by the Danish health authorities’ recommendations for prenatal care [33,34]. These groups include pregnant women with severe social challenges, previous or current severe mental health conditions, previous or current harmful use of addictive substances such as drugs or alcohol, or uncertainties about the parents’ capability to care for the child.

#### 2.1.1. Inclusion and Exclusion Criteria

We included pregnant women who met the following criteria: at least 15 years of age, residing in Odense Municipality, classified by prenatal care group 3 or 4 and enrolled at the family clinic at Odense University Hospital. Participants were excluded if they were unable to complete questionnaires in Danish or English, had a life-threatening illness affecting the parent or child, or had participated in the FACAM project with an older child. Twin pregnancies were excluded due to their distinct pregnancy and parenting demands, and women were withdrawn if their child was subsequently placed in out-of-home care.

#### 2.1.2. Recruitment

We identified 562 women as eligible for the study through a screening of visitation lists from the family clinic. During their first pregnancy visit to the clinic, midwives invited these women to participate in the study. Participants received both oral and written information about the study and provided written consent, either immediately or at a subsequent appointment. At this initial visit, the midwife also categorized each woman into one of four levels of concern: (1) high concern (the presence of a prior or current report to child protective services about the family), (2) medium concern (a high likelihood of a report to child protective services being made during pregnancy), (3) low concern (the family might benefit from an attachment-based course but otherwise presented few concerns), and (4) minimal to no concern (little to no concern about the family’s development or well-being). The categorization was based on information gathered through conversation with the woman, patient-reported data, and medical records. This categorization informed the type and intensity of intervention provided to each participant.

### 2.2. Randomization and Blinding

After providing informed consent, each participant was instructed by a research team member to complete the baseline questionnaire. Once completed, randomization (1:1) was conducted to assign participants to either the FACAM intervention group or the CAU group. The randomization process utilized the “OPEN randomize” tool within REDCap, which included a randomization sequence generated by an independent data analyst. Participants were stratified into two groups based on their level of concern as assessed during the first pregnancy visit: high concern (levels 1 and 2), and low concern (levels 3 and 4).

**Figure 1:**
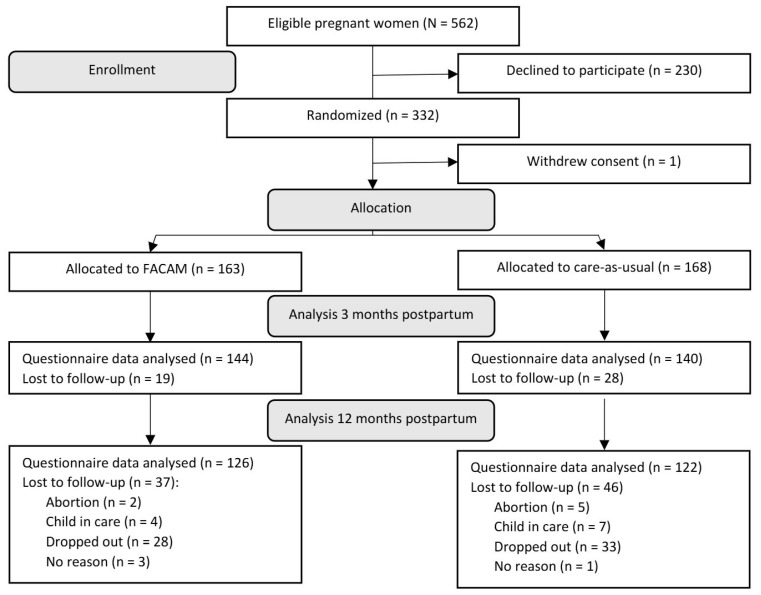
Flow chart of participant inclusion, allocation, and analysis. Notes: FACAM: intervention group.

In total, 331 pregnant women were randomized into the study, with 163 assigned to FACAM and 168 to CAU. The participant flow is illustrated in Figure 1.

Following randomization, a research team member informed both the participant and the municipality’s project coordinator of the assigned group. For those allocated to the intervention group, a FACAM person was assigned based on the geographical location of the family, ensuring accessibility and continuity of care. The intervention commenced immediately after group assignment.

Blinding participants and care providers to group allocation was not feasible due to the open nature of the intervention. However, coders and data analysts remained blinded to allocation status to minimize bias during data processing and analysis.

### 2.3. Intervention and Comparison

#### 2.3.1. Care as Usual

Participants in the control group received standard care, which is freely provided by public health services in Denmark. This includes prenatal care, birth assistance, baby health checkups, and support from social services. For women classified in care groups 3 and 4, routine prenatal care typically involves four to eight midwife consultations, three general practitioner appointments, and two ultrasound scans [33,34,35]. Based on individual needs, a specialist team at the family clinic provides additional checkups or services, such as consultations with obstetricians, extra ultrasounds, or meetings with social workers or therapists [33,34]. Furthermore, the health visitor can arrange a visit during pregnancy [36].

Uncomplicated births are managed by midwives in hospital settings, with obstetricians involved if complications arise. Upon discharge, the midwife notifies the family’s municipality of residence about the birth. According to guidelines from the Danish National Board of Health, all families are offered five standard home visits by health visitors. These visits are typically initiated within seven days postpartum. Families with additional needs may receive extra home visits or tailored parenting support. In Odense Municipality, health visitors screen for symptoms of depression once during pregnancy and again after birth. In addition to home visits, mothers are offered a postbirth check-up with their general practitioner and three well-child check-ups during the child’s first year of life [36].

#### 2.3.2. The FACAM Intervention

Participants in the intervention group received standard care along with the FACAM intervention. The intervention was developed between 2017 and 2018 through a collaboration between Odense Municipality and Odense University Hospital. Odense, Denmark’s third-largest city, has approximately 210,000 inhabitants and a diverse population across socioeconomic, cultural, and ethnic backgrounds.

The FACAM intervention was created to meet the need for an early, interdisciplinary approach to support pregnant women in vulnerable positions and their families. The intervention focused on strengthening mother–child attachment, promoting parental mental health and caregiving capacity, and improving access to social support. It was developed by a multidisciplinary group of practitioners and project leaders, with support from leaders from both municipality and hospital sectors as well as researchers. The content drew on research, evaluation reports from earlier initiatives [37], and input from citizens and frontline staff. The intervention was implemented in October 2018.

A central feature of the FACAM intervention was the assignment of a dedicated support person (FACAM person) to each participant. This professional provided continuous support from pregnancy through the child reached school age, offering stability, personalized guidance, and easy access to care. The intervention was guided by principles of mentalization [38,39]. It also incorporated strategies to reduce health inequalities [40,41], drawing on frameworks that emphasize early, relational, and cross-sectoral support for families in vulnerable positions. In addition, the intervention integrated evidence from midwifery research highlighting the benefits of continuity in care and stable caregiver relationships [42]. The overarching aim was to reduce stress and practical burdens so families could focus on parenting and child well-being.

FACAM persons were either trained health visitors or family therapists. Health visitors are registered nurses with specialized training (1.5 years) in maternal, child, and family health while family therapists typically had backgrounds in early childhood education, social work, or psychology, supplemented by therapeutic training. All FACAM persons completed a four-day training program in mentalization, along with short courses on topics such as mental health and interprofessional collaboration. They received ongoing supervision from a clinical psychologist. In addition, health visitors were trained in the Alarm Distress Baby Scale (ADBB) [43] for assessing early signs of infant social withdrawal.

Assignment of FACAM persons was based on the family’s assessed level of concern. Participants categorized as low concern were assigned a health visitor who also served as their standard municipal health visitor. Families with higher levels of concern were assigned a family therapist as their FACAM person, while routine home visits were conducted by a health visitor who might not have received FACAM training.

Although the intervention did not follow a strict manual, it was guided by flexible protocols tailored to individual family needs (guidelines in Danish are available upon request). These protocols outlined key responsibilities and suggested contact frequency. FACAM persons provided support with well-child check-ups, vaccinations, daycare registration, contraception, women’s health, parenting, family dynamics, healthcare, and work–life balance. They also facilitated referrals to services for financial, employment, or education support as needed.

FACAM support was typically provided through home visits or meetings at municipal health or family centers. Each FACAM person participated in at least one midwifery consultation during pregnancy, facilitating interdisciplinary collaboration and ensuring a smooth transition from hospital to municipal care. When necessary, the FACAM person coordinated with other professionals and accompanied families to external appointments, effectively acting as case managers who helped families navigate the care system [44]. To promote coordination and integration, FACAM persons and professionals from both sectors met regularly in joint sessions to build a shared understanding across disciplines and promote relational coordination. The intervention aimed to deliver integrated care across several dimensions: person-centered care, professional and normative integration, and, partly, organizational integration [45].

This paper focuses on the early phase of the FACAM intervention, encompassing pregnancy and the child’s first year of life. During this period, participants could receive up to 47 hours of support from their FACAM person. Additionally, all participants in the FACAM intervention group were offered an attachment-based parenting course. As described earlier, participants were categorized into four levels of concern during recruitment. Those identified as low concern (level 3 or 4) were invited to participate in eight two-hour sessions of the Circle of Security Parenting (COS-P) group program [46] when their child was approximately two months old. Participants in the high-concern categories (level 1 or 2) were offered up to 50 hours of individualized sessions, delivered from pregnancy until the child was two months old. The sessions were grounded in attachment theory [47] and included, among other elements, a locally developed pregnancy interview focusing on the emerging parent–child relationship.

Participants in both groups could receive supplementary care during the trial. The FACAM intervention was discontinued if the participant did not wish to continue, if they relocated from Odense Municipality, or if the child was placed in out-of-home care.

### 2.4. Data Collection

We collected data from participants through questionnaires at four time points, twice during pregnancy (T0 baseline part 1 before randomization and T1 baseline part 2 at approximately gestational week 25) and twice after the child was born (T2 3 months postpartum, and T3 12 months postpartum).

Questionnaires were distributed via a secure online survey platform, REDCap, hosted on OPEN storage at Odense University Hospital, Region of Southern Denmark. Participants were contacted through Digital Post, a secure digital mailbox system used by Danish citizens, which included a link to the questionnaire. Participants who did not respond received up to three automated reminders, followed by manual follow-up to encourage completion. If needed, women could receive phone support to assist with completing the questionnaire. To optimize participation rates, all women received a gift card worth DKK 200 (EUR ~25) after completing the T1, T2, and T3 questionnaires.

Data storage and analysis were conducted using secure servers provided by The Agency for Governmental IT Services (Statens IT). Access to the full dataset was restricted to the trial statisticians, the principal investigator, and the co-principal investigator, ensuring data security and confidentiality.

### 2.5. Measures and Outcomes

The timing of baseline measures and outcomes analyzed in this paper is presented in Table 1. Further details on all measures can be found in the protocol paper [48].

#### 2.5.1. Baseline Measures

At baseline, participants provided self-reported information about maternal demographics and background, including age, education, occupation, language spoken at home, number of children, household status, substance use, and breastfeeding expectations. Baseline assessments (T0 and T1) also included the following scales: Prenatal Parental Reflective Functioning Questionnaire (P-PRFQ) [49], which measures maternal reflective functioning during pregnancy, the Hospital Anxiety and Depression Scale (HADS) [50,51], which measures symptoms of anxiety and depression, and the Experiences in Close Relationship Scale-Short Form (ECR-S) [52], which assesses attachment-related anxiety and avoidance. Additionally, the Short Warwick-Edinburgh Mental Wellbeing Scale (SWEMWBS) [53,54], designed to assess mental well-being, was included at baseline and later used as an outcome measure at T2 and T3.

At T1 (gestational week 25), participants completed additional assessments focused on trauma and PTSD symptoms. These included the PTSD-8 [55], a brief measure for assessing symptoms of post-traumatic stress disorder, and the Adverse Childhood Experiences (ACE-10) questionnaire [56], which captures information on childhood trauma and adversity.

#### 2.5.2. Outcomes Analyzed in This Paper

The primary outcome of the trial, maternal sensitivity, was assessed through video recordings at 3 and 12 months postpartum, coded using the Coding Interactive Behavior (CIB) instrument [57]. The results of this outcome, along with those assessing child well-being and development, have been reported in a separate publication [32].

This paper focuses on secondary outcomes related to maternal mental health and parental functioning. These outcomes were assessed at 3 months postpartum (T2) and/or 12 months postpartum (T3). Some measures were administered at both time points, while others were collected only once. The following measures were included:

Short Warwick-Edinburgh Mental Wellbeing Scale (SWEMWBS) [53,54]: A 7-item measure assessing maternal mental health. Total scores range from 7-35, with higher scores indicating better well-being. Cronbach’s alpha was 0.83 at T2 and 0.84 at T3.

Edinburgh Postnatal Depression Scale (EPDS) [58]: A 10-item measure assessing symptoms of postpartum depression. Total scores range from 0 to 30, with higher scores indicating more symptoms. A cut-off of ≥11 indicates clinically significant symptoms, based on the Danish validation [59]. Cronbach’s alpha was 0.87 at T2 and 0.88 at T3.

Being a Mother (BaM-13) [60]: A 13-item measure evaluating a mother’s experience and satisfaction with motherhood. Total scores range from 0 to 39, with lower scores indicating greater satisfaction. Cronbach’s alpha was 0.82.

Parental Reflective Functioning Questionnaire (PRFQ) [61]: An 18-item measure of parental reflective functioning, including three subscales: Pre-Mentalizing Modes (PRFQ-PM, lower scores indicate better function), Certainty About Mental States (PRFQ-CMS, higher scores indicate better function (recoded so middle value is scored high)), and Interest and Curiosity in Mental States (PRFQ-IC, higher scores indicate better function). Cronbach’s alphas were 0.56 (PM), 0.66 (CMS), and 0.71 (IC).

Parenting Stress Scale (PSS) [62,63]: An 18-item measure of parenting stress with two subscales: Parental Stress (PS) and Lack of Parental Satisfaction (LPS). Items 2 and 11 are excluded from the subscale scores and LPS items are reversed. The summed subscale scores range from 1 to 45 for PS and 1 to 35 for LPS, with lower scores indicating less stress and greater satisfaction. Cronbach’s alphas were 0.82 for PS and 0.80 for LPS.

In addition to these scales, several outcomes were assessed using single-item measures assessing smoking behaviors, maternal health, maternal life satisfaction, feelings of loneliness, access to social and practical support, employment, breastfeeding, and concerns about housing, employment, and relationships.

### 2.6. Fidelity

We documented whether each FACAM participant was assigned to a health visitor or a family therapist. Following each visit, the FACAM person filled out a brief questionnaire detailing the type of support offered to the family. Additionally, we recorded attendance at the attachment sessions.

### 2.7. Sample Size Justification

The study was powered to detect an average effect size of 0.33 for the primary outcome parental sensitivity [64]. Due to high dropout rates for video observations (194 participants (103 FACAM and 91 CAU)), the power was reduced to 63%. While no formal power calculation was performed for secondary outcomes, the sample included in this paper, based on questionnaire data (248 responses: 126 FACAM and 122 CAU), provided a 74% power to detect a similar effect size of 0.33, assuming normally distributed outcomes and a two-sided alpha of 0.05.

### 2.8. Statistical Analyses

We tested all outcomes using linear regression with robust standard errors to estimate mean differences and account for potential heteroscedasticity in the error term. The treatment effect was estimated using a binary treatment indicator, and variables showing significant differences (*p* < 0.05) between the intervention and CAU groups at baseline were included as control variables. All tests were two-sided with a significance level of 0.05 [65].

The main analysis followed the intention-to-treat (ITT) principle, including all participants in the arm to which they were originally allocated, regardless of the extent of treatment received. Missing data were handled using multiple imputation, performed separately for each group and incorporating all available baseline data. We did not impute observations for seven participants whose pregnancies were aborted, resulting in a total of 324 participants in the imputed dataset. This method assumes data are missing at random (MAR) or missing completely at random (MCAR) [66]. To test the MCAR assumption informally, we used logistic regression models to assess whether baseline characteristics could predict missingness at the 3- and 12-month postpartum data collection points (test not shown). Of the 42 baseline characteristics examined, only three were significant at the 5% level in both data rounds. Additionally, joint significance tests did not indicate significant prediction of missingness at the 5% level. The analysis found that highly educated mothers, mothers with fewer worries about their job situation, and mothers who felt more depressed were less likely to be missing in the survey rounds. Based on this we were confident assuming that the data were missing at random.

Sensitivity analyses were conducted to evaluate the robustness of our findings. These analyses included a complete case analysis and an instrumental variable (IV) approach to account for non-participation in the intervention.

#### Subgroup Analyses

We conducted subgroup analyses to explore potential differences in treatment effects across various participant subsets. These subsets included education level (high school or less versus more than high school), level of concern about the family (level 1/2 versus level 3/4), trauma level (ACE < 3 versus ACE ≥ 3), and attendance (dose received). Subgroup analyses were performed using interaction models, which incorporated both the subgroup characteristic and an interaction term between the treatment indicator and the subgroup indicator into the baseline regression model. The coefficient of the interaction term indicated whether the treatment effect differed significantly across subgroups.

Due to data limitations, we were unable to perform subgroup analyses for certain characteristics, including parity (primiparous versus multiparous), type of provider (health visitor versus family therapist), adult attachment style (measured by ECR-S), initial reflective functioning level (lowest 50% versus highest 50%), and initial levels of depression or anxiety (levels above cut-off and below cut-off).

## 3. Results

During the recruitment period from October 2018 to August 2021, 562 pregnant women were invited to participate in the trial. Of these, 332 women (57%) provided informed consent and completed the baseline questionnaire. One participant later withdrew consent, leading to the deletion of her data. This resulted in a final baseline sample of 331 participants, with 163 women allocated to the FACAM intervention group and 168 to the CAU group (se Figure 1).

### 3.1. Participant Characteristics

Table 2 provides an overview of the descriptive statistics for the study population, categorized by allocation group. Baseline differences were minimal, with only two significant variations observed: a greater proportion of women in the control group were expecting their first child (66% compared to 56%) and a greater proportion were in education (23% compared to 14%). On average, participants were 30 years old, and 82% were living with a partner. Nearly half of the participants had completed high school or less as their highest level of education. Employment statistics showed that 36% were employed, 17% were on sick leave, and 28% were not engaged in employment or education. Participants reported an average of 2.5 traumatic childhood events, and 20% exhibited indications of posttraumatic stress disorder (PTSD). Participants in the higher-concern group (N = 91) exhibited greater risks across several factors, including younger age, lower education levels, reduced employment rates, higher smoking rates, and increased levels of childhood trauma, and fewer had never regularly used drugs compared to those in the lower-concern group (see Supplementary Table S1).

### 3.2. Participation

The FACAM intervention included two components: visits from the FACAM person and attachment-based sessions. On average, participants in the FACAM group received 9.3 contacts (median 7) with their FACAM person by the time their child turned 12 months. Of these, 33% occurred during pregnancy and 67% during the first year postpartum. Participants assigned a family therapist as their FACAM person had more contacts on average (15 visits) compared to those with a health visitor (5.7 visits). However, 16% of participants (26 women) had no recorded contact with their FACAM person, which could be attributed to factors such as health visitor changes, a lack of perceived need, or participants declining the intervention.

Attendance at attachment sessions was lower than anticipated, with only 25 participants attending the Circle of Security Parenting program and 51 attending individual attachment sessions (4 participants received both interventions). Over half of the participants (55%, or 91 participants) did not participate in any attachment sessions, partly due to implementation challenges, including disruptions caused by COVID-19 restrictions.

In total, participants received an average of 15 contacts through the FACAM intervention (combining FACAM visits and attachment sessions). As expected, participants in the high-concern group had more contacts on average (23) compared to those in the low-concern group (12).

### 3.3. Attrition

At baseline, 331 participants completed the questionnaire. This number decreased to 284 at 3 months postpartum and further to 248 at 12 months postpartum (see Figure 1 and Table 3). Dropout rates for questionnaire data were relatively consistent between the FACAM and CAU groups. However, families categorized as medium or high concern were more likely to drop out compared to those in the low-concern group. While the two groups were initially well-balanced at baseline in terms of background characteristics, differences emerged at later follow-ups due to selective dropout and the higher attrition rate in the CAU group. These differences were accounted for in the regression analyses. Participants were lost to follow-up for various reasons, including withdrawal from the study, children being placed in out-of-home care, relocation to a different municipality, or passive dropout, where participants did not actively withdraw but failed to complete the questionnaire despite multiple follow-up attempts.

### 3.4. Effect Analysis

The means and standard deviations for the outcomes analyzed in this paper are presented in Table 4.

Table 5 presents the regression results comparing mothers in the FACAM group with those receiving CAU.

At T2 3 months postpartum, we did not find any statistically significant differences between the groups. While fewer FACAM mothers reported smoking in the house at 3 months postpartum, this result was only borderline significant and did not persist at 12 months postpartum. At T3 12 months postpartum, FACAM mothers reported greater concern about housing issues (b = 0.56 [0.06, 1.06], *p* = 0.03). We did not find any other significant differences between the groups for the reported outcomes.

### 3.5. Subgroup Analysis

We investigated whether the effects of the intervention varied across subgroups defined by three outcomes: level of education, level of concern, and level of trauma (Supplementary Table S2). The analysis did not reveal any significant interactions, indicating that the effect of FACAM was consistent across these subgroups.

For FACAM families, we also explored whether there were any differential effects related to participation, measured by the number of intervention sessions attended. The estimates in this analysis should be interpreted as the incremental change in outcomes associated with an additional FACAM contact. However, the number of contacts is not random; it may depend on factors such as family needs and other characteristics. As a result, these findings should not be interpreted causally. The analysis did not reveal any significant interactions, suggesting that the effect of FACAM was consistent regardless of the level of participation in the intervention (analysis not shown).

### 3.6. Sensitivity Analysis

We conducted sensitivity analyses, including a complete case regression (without imputation) and an instrument variable (IV) estimation (see Supplementary Table S3).

The complete case analysis revealed no significant differences between groups, while the IV analysis indicated two notable findings: a reduction in smoking in the household at 3 months postpartum among FACAM mothers and an increase in housing-related worries at 12 months postpartum compared to CAU mothers.

As these findings are based on single-item questions rather than scales, and only two significant results emerged out of 68 tests, they may be attributed to chance. However, there is some consistency across the three types of analyses that supports these observations. The effect size for reduced smoking in the household was 0.23, indicating a potential small reduction at 3 months postpartum, with the primary and complete case analyses yielding marginal *p*-values (*p* = 0.06 and *p* = 0.05, respectively). This effect, however, was not observed at 12 months postpartum.

The effect size for increased housing-related worries was 0.49, suggesting a potential medium-sized increase, with the primary analysis yielding a marginal *p*-value (*p* = 0.06). Notably, this finding was not replicated in the complete case analysis.

Although the sensitivity analysis highlighted two potential findings, overall, it supported the primary regression analysis, confirming that the FACAM intervention did not have significant effects on the studied outcomes.

## 4. Discussion

This paper examined the effects of the interdisciplinary FACAM intervention, designed for pregnant women in vulnerable positions, on maternal mental health and parental functioning during the first year of motherhood.

At 3 months postpartum, we observed a potential temporary reduction in smoking in the house, and at 12 months postpartum, FACAM mothers reported greater concern about housing issues. However, as only one significant outcome emerged from the effect analysis, it is possible that this result occurred by chance.

The lack of clear effects of the FACAM intervention is consistent with the findings from the primary analysis reported in a previous paper, which examined maternal sensitivity and parent–child interaction outcomes [32]. In that analysis, we found that CAU children were significantly more involved in interactions than FACAM children at 4–6 months old (b = −0.25, [−0.42; −0.08], d = −0.42). However, this result may have been influenced by biased dropout, as families in the control group with higher vulnerability levels were more likely to discontinue participation.

Overall, the results suggest that the FACAM intervention was not superior to CAU in terms of maternal mental health, parental functioning, child development, or parent–child interaction outcomes.

These findings diverge from results reported in several systematic reviews, which generally report positive effects of early parenting interventions—particularly among at-risk families. For example, structured interventions for families with infants have shown improvements in maternal sensitivity, reductions in depressive symptoms, and better parent–child relationships [19,22,24]. Attachment- and mentalization-based interventions have also demonstrated reductions in disorganized attachment and, in some cases, improved reflective functioning [23,25]. Although effect sizes are often modest and vary across studies, the overall pattern in the literature is more encouraging than what we observed in the FACAM trial. Differences in intervention intensity, timing, implementation, and contextual factors such as Denmark’s strong baseline of universal care may help explain these discrepancies.

### 4.1. Challenges in Implementation and Participation

One explanation for the lack of intervention effects is that participants, particularly those in the low-concern group, received less intervention than anticipated. On average, participants in the low-concern group participated in an average of 5.7 visits with the FACAM person compared to 15 visits for the high-concern group. Attendance at attachment sessions was also lower than anticipated, with only 25 participants in the low-concern group attending the offered group intervention. Focus group interviews with FACAM persons revealed that some professionals felt the additional care was unnecessary for certain families or that contacts were too frequent [67]. As a result, some visits were replaced with phone calls or omitted altogether. These experiences illustrate the ethical and practical challenges of conducting an RCT of a comprehensive intervention in a setting where interventions must be both meaningful to participants and respectful of their time priorities during the critical period of early family formation.

### 4.2. Reflections on Recruitment and Retention

A major factor influencing these findings was the difficulty in recruiting and retaining a sufficient number of families with high levels of concern, who stood to benefit most from the intervention. Initially, the study aimed to recruit an equal number of participants with high (levels 3 + 4) and low (levels 1 + 2) levels of concern. However, only 27% of the recruited participants belonged to the high-concern group. Additionally, a substantial number of control group participants with high levels of concern withdrew from the study. These challenges underscore the difficulty of engaging families with higher complexity in research and interventions. It is well-established that this population may have less capacity to sustain participation over time.

To address the recruitment challenges, the recruitment strategy was revised midway through the trial. Initially, midwives provided preliminary information to potential participants, followed by phone contact with researchers to explain the study and obtain consent. This approach was replaced with a more direct strategy, where trained midwives provided all study information and obtained consent on-site, with researchers available for follow-up questions. While this adjustment improved recruitment rates, many potential participants still declined to join the study. Common reasons included a perceived lack of need or interest in additional support if assigned to the intervention group, as well as concerns about the time demands of the intervention. Some participants preferred to remain in standard care to avoid feeling stigmatized, while others favored continuing care with professionals from previous pregnancies who were not involved in the current study.

### 4.3. Reflections on Intervention Design and Feasibility

In retrospect, more extensive feasibility testing during the planning phase could have provided valuable insights into the needs and preferences of the target group. Initial feasibility efforts included interviews with stakeholders to better understand the needs of families in vulnerable positions. While these interviews provided useful information, broader input through a dedicated user panel would have been beneficial. Efforts to establish a user panel during the planning phase were unsuccessful due to logistical challenges and time constraints, but a longer feasibility phase might have clarified recruitment challenges and the needs of both low- and high-concern families.

Families classified with the lowest level of concern (level 1) were assigned a health visitor as their FACAM person. Over the course of the trial, it became evident that these families required minimal additional support, as health visitors felt confident in meeting their needs through standard care. Consequently, after the trial’s conclusion, the FACAM intervention was revised to focus exclusively on families with higher levels of concern. In this revised model, FACAM persons have a less comprehensive role but continue to conduct the attachment sessions. This adjustment ensures that families with high needs can continue to have access to early and timely support without waiting for additional assistance through standard social welfare procedures.

Following the trial, when the revised FACAM intervention was implemented, it was offered to all families within the newly defined target group (levels 3 and 4). By removing the requirement for families to consent to randomization, participation rates increased significantly. This change underscores the challenges that families facing adversity may encounter in understanding and accepting randomization and other aspects of trial methodology [68].

Research indicates that participant information sheets are often overly lengthy and complex, making them difficult for many participants to fully comprehend [69,70]. This issue is particularly pronounced among vulnerable populations, where trial participants may struggle to process the information provided [71,72]. A key contributing factor is that such materials are often driven by regulatory requirements rather than tailored to meet participants’ needs [73]. Randomization, in particular, is a concept that only about half of participants understand adequately [74], and comprehension rates are even lower in populations with greater vulnerability [75].

To overcome these barriers, we prioritized making participant information materials as accessible as possible [76]. We used simple and clear language, provided concise and relevant details, and offered oral explanations from both frontline staff and research team members. To further support participants, we developed supplementary resources, including a short informational video featuring the trial’s principal investigator. These efforts likely played a critical role in improving participation and retention rates. Without such initiatives, engaging and retaining families in the study would likely have been even more challenging. Interestingly, families in the intervention group, who had more frequent contact with professionals through the FACAM program, demonstrated higher retention rates compared to those in the CAU group. This suggests that the additional professional support provided by the intervention may have helped sustain participant engagement, even though the overall intervention effects were not significant.

### 4.4. Broader Context and Implications

As highlighted in our paper on the primary analysis of maternal sensitivity [32], one key reason for the lack of significant differences observed in this study could be the high standard of care provided in Denmark. The comparison in this study was not between treated and untreated groups but between two forms of intervention, both delivered within Denmark’s well-established welfare system. Denmark’s healthcare infrastructure offers comprehensive, standardized care, including prenatal and postnatal care, midwifery services, and early childhood programs. Welfare-oriented policies, such as universal healthcare, generous parental leave, subsidized childcare, and robust social support systems, create an environment that inherently supports pregnant women and families. This high baseline level of care reduces the scope for additional interventions to demonstrate measurable effects even though the intervention is well-designed and implemented. Similar findings have been observed in other Danish trials, including studies by Røhder et al. [77], Pontoppidan et al. [78,79], Brixval et al. [80], and Stuart et al. [81].

The study context highlights the need to interpret research findings with nuance. While the robust healthcare and social support systems in Denmark limit the potential for additional interventions to produce measurable effects, this does not diminish the importance of developing and testing programs that address specific unmet needs or gaps in care for vulnerable populations. The study’s transferability to countries with less developed health or social care systems would require cultural and structural adaptation through local co-construction of the intervention. Targeted interventions remain crucial to ensuring that no families are left behind, even in settings with high-quality universal care.

### 4.5. Study Limitations

This study has several limitations. Participation was limited to women who were able to complete questionnaires in Danish or English, which may affect generalizability. Although the intervention was designed for families in vulnerable positions, participants with more severe challenges were underrepresented, limiting the study’s ability to detect effects among the target group. Additionally, there were notable dropout rates, particularly among participants with higher levels of concern in the control group, which may have introduced bias and affected the interpretation of outcomes.

## 5. Conclusions

We examined the effects of the interdisciplinary FACAM intervention on maternal mental health and parental functioning and found no evidence that the FACAM intervention was superior to standard care at 3 and 12 months postpartum. Given that the control group received high-quality standard care, comparable to the FACAM intervention, we conclude that FACAM does not demonstrate superiority over usual care in this context. These findings reflect the challenges of detecting additional effects of interventions in welfare systems like Denmark, where a strong baseline of care is universally available. Recruitment challenges and underrepresentation of high-need families may have also influenced the results. Future studies should consider optimizing recruitment strategies and evaluating how interventions can better address the specific needs of vulnerable populations within a high-quality care setting.

## Figures and Tables

**Table 1:** Overview of the timing of measure administration across the study period.

Measures	Measure/Content	T0	T1	T2	T3
Sociodemographic measures	Age, household status, etc.	√		√	√
Well-being	SWEMWBS	√		√	√
Anxiety and depression	HADS	√			
Parent health and satisfaction	Health and satisfaction	√		√	√
Breastfeeding duration				√	√
Education and job expectation		√			√
Network	Confidants, loneliness, support	√		√	√
Worry	Housing, employment, relationship	√		√	√
Alcohol, drugs, and medicine use		√			
Smoking		√		√	√
Experiences in close relationships	ECR-S	√			
Prenatal parental reflective functioning	P-PRFQ		√		
PTSD symptoms	PTSD-8		√		
Childhood trauma	ACE10		√		
Postnatal depression	EPDS			√	√
Being a mother	BaM-13			√	
Parental reflective functioning	PRFQ				√
Parental stress	PSS				√

Notes: T0: baseline part 1 before randomization, T1: baseline part 2 at approximately gestational week 25, T2: 3 months postpartum, T3: 12 months postpartum. SWEMWBS: Short Warwick-Edinburgh Mental Wellbeing Scale, HADS: Hospital Anxiety and Depression Scale, ECR-S: Experiences in Close Relationship Scale-Short, P-PRFQ: Prenatal Parental Reflective Functioning Questionnaire, PTSD-8: Post Traumatic Stress Disorder Inventory, ACE10: Adverse Childhood Experiences, EPDS: Edinburgh Postnatal Depression Scale, BaM-13: Being a Mother, PRFQ: Parental Reflective Functioning Questionnaire, PSS: Parental Stress Scale.

**Table 2:** Study population characteristics at baseline and comparison between groups.

	Type	CAU N = 168		FACAM N = 163		Difference	
		M/P	SD	M/P	SD	T-Stat	*p*
Mother age (year)	M	29.64	(5.65)	29.64	(5.43)	0.01	0.99
Mother health	M	7.14	(1.76)	7.13	(1.84)	0.07	0.94
Mother life satisfaction	M	7.79	(1.85)	7.67	(1.88)	0.60	0.55
SWEMWBS well-being	M	23.70	(3.81)	23.81	(4.35)	−0.24	0.81
Loneliness ¤	M	2.41	(0.92)	2.58	(0.93)	−1.70	0.09
Access to practical help	M	4.10	(1.02)	4.00	(1.05)	0.84	0.40
Access to somebody to talk to	M	4.59	(0.79)	4.48	(0.88)	1.14	0.26
HADS anxiety ¤	M	6.81	(3.90)	6.76	(3.81)	0.12	0.91
HADS depression ¤	M	4.15	(3.01)	4.64	(3.53)	−1.36	0.18
PTSD total score ¤	M	13.83	(5.82)	14.01	(6.16)	−0.28	0.78
ECR fear of abandonment ¤	M	19.64	(7.09)	18.30	(6.95)	1.73	0.08
ECR fear of intimacy ¤	M	14.01	(6.88)	13.17	(6.49)	1.13	0.26
P-PRFQ opacity of mental states	M	4.60	(1.22)	4.57	(1.32)	0.21	0.83
P-PRFQ reflecting on the fetus-child	M	5.11	(1.04)	4.98	(1.01)	1.10	0.27
P-PRFQ the dynamic nature of mental states	M	4.56	(1.05)	4.35	(1.14)	1.75	0.08
ACE total score ¤	M	2.46	(2.26)	2.40	(2.20)	0.21	0.83
Units of alcohol prior to pregnancy	M	1.62	(2.26)	1.39	(1.97)	0.97	0.33
Units of alcohol during pregnancy	M	0.01	(0.11)	0.02	(0.19)	−0.74	0.46
Expecting first child	P	0.68	(0.47)	0.56	(0.50)	2.15	0.03
Cohabit with partner	P	0.81	(0.39)	0.83	(0.38)	−0.44	0.66
Only speak Danish at home	P	0.83	(0.37)	0.81	(0.39)	0.56	0.58
High school or less	P	0.41	(0.49)	0.42	(0.49)	−0.12	0.91
Vocational or secondary education	P	0.19	(0.39)	0.23	(0.42)	−0.82	0.42
College, bachelor, tertiary, or longer education	P	0.40	(0.49)	0.36	(0.48)	0.80	0.42
Employed	P	0.36	(0.48)	0.36	(0.48)	−0.09	0.93
Sick leave	P	0.14	(0.35)	0.20	(0.40)	−1.30	0.20
Unemployment benefit	P	0.07	(0.26)	0.06	(0.23)	0.60	0.55
Social assistance/unemployment program	P	0.13	(0.34)	0.20	(0.40)	−1.75	0.08
In education	P	0.23	(0.42)	0.14	(0.35)	2.13	0.03
Unemployment no benefits	P	0.03	(0.17)	0.01	(0.08)	1.61	0.11
Smoking regularly	P	0.11	(0.32)	0.13	(0.34)	−0.60	0.55
Never regularly used drugs like hash, pot, marihuana	P	0.89	(0.31)	0.86	(0.35)	0.94	0.35
Never regularly used drugs like amphetamine, ecstasy, cocaine, LSD	P	0.95	(0.21)	0.94	(0.24)	0.55	0.58
Medicine during pregnancy (including non-prescription painkillers)	P	0.60	(0.49)	0.62	(0.49)	−0.45	0.65
Expect to breastfeed	P	0.96	(0.19)	0.97	(0.18)	−0.22	0.83

Notes: CAU: Care as usual, FACAM: Intervention, M: Mean, P: Proportion, SD: Standard deviation, T-Stat: T statistic, *p*: *p*-value, ¤: Low score is best.

**Table 3:** Questionnaire response rates over time by group and level of concern.

	Responses			CAU		FACAM	
Time	Total N (%)	Low n (%)	High n (%)	Low n (%)	High n (%)	Low n (%)	High n (%)
T0	331	240	91	122	46	118	45
T1	312 (94)	229 (95)	83 (91)	116 (95)	42 (91)	113 (96)	41 (91)
T2	284 (86)	218 (91)	66 (73)	110 (90)	30 (65)	108 (92)	36 (80)
T3	248 (75)	196 (82)	52 (57)	98 (80)	24 (52)	98 (83)	28 (62)

Notes: CAU: care as usual, FACAM: intervention, Low/High: level of concern, T0: baseline part 1 before randomization, T1: baseline part 2 at approximately gestational week 25, T2: 3 months postpartum, T3: 12 months postpartum.

**Table 4:** Means and standard deviations for maternal questionnaire outcomes at baseline, 3 months, and 12 months postpartum by group.

	T0				T2				T3			
	FACAM N = 163		CAU N = 168		FACAM N = 144		CAU N = 140		FACAM N = 126		CAU N = 122	
	M/P	SD	M/P	SD	M/P	SD	M/P	SD	M/P	SD	M/P	SD
SWEMWBS well-being	23.70	(3.81)	23.81	(4.35)	24.20	(3.85)	24.08	(3.78)	24.48	(3.69)	24.23	(4.09)
Mother health	7.14	(1.76)	7.13	(1.84)	7.57	(1.76)	7.39	(1.86)	7.22	(2.05)	7.40	(1.97)
Mother life satisfaction	7.79	(1.85)	7.67	(1.88)	8.41	(1.60)	8.11	(1.84)	7.94	(2.11)	7.94	(1.91)
Loneliness ¤	2.41	(0.92)	2.58	(0.93)	2.29	(1.02)	2.35	(1.04)	2.13	(1.02)	2.32	(0.95)
Access to practical help	4.10	(1.02)	4.00	(1.05)	3.81	(1.18)	3.84	(1.11)	3.80	(1.08)	3.74	(1.09)
Access to somebody to talk to	4.59	(0.79)	4.48	(0.88)	4.58	(0.69)	4.61	(0.67)	4.57	(0.78)	4.52	(0.83)
Smoking regularly ¤ (P)	0.11	(0.32)	0.13	(0.34)	0.14	(0.34)	0.13	(0.34)	0.16	(0.36)	0.16	(0.37)
Smoking in house ¤ (P)	0.08	(0.28)	0.10	(0.30)	0.03	(0.17)	0.00	(0.00)	0.02	(0.16)	0.02	(0.13)
Employed (P)	0.36	(0.48)	0.36	(0.48)					0.48	(0.50)	0.44	(0.50)
BaM-13 Being a mother ¤					10.67	(6.06)	10.78	(5.68)				
Worries: Housing ¤					2.57	(2.11)	2.31	(1.83)	2.19	(1.67)	2.51	(1.99)
Worries: Employment ¤					3.05	(2.00)	2.99	(2.08)	3.45	(2.21)	3.43	(2.12)
Worries: Relationship ¤					2.74	(1.91)	2.64	(1.93)	2.72	(2.07)	2.73	(1.95)
EPDS Postnatal depression ¤					7.54	(4.49)	8.03	(5.25)	8.16	(5.36)	7.66	(4.92)
Started to breastfeed (P)					0.96	(0.20)	0.92	(0.27)				
Still breastfeeding (P)					0.63	(0.48)	0.66	(0.48)	0.31	(0.47)	0.35	(0.48)
PSS PS ¤									19.77	(5.84)	20.20	(5.98)
PSS LPS ¤									9.30	(3.09)	9.29	(2.79)
PRFQ PM ¤									10.12	(4.28)	9.50	(3.07)
PRFQ CMS									30.43	(5.66)	28.94	(6.57)
PRFQ IC									36.43	(4.56)	36.28	(4.55)

Notes: T0: baseline part 1 before randomization, T2: 3 months postpartum, T3: 12 months postpartum, CAU: care as usual, FACAM: intervention, M: mean, P: proportion, SD: standard deviation, ¤: low score indicates better outcomes.

**Table 5:** Regression results comparing FACAM and CAU for maternal outcomes at 3 and 12 months postpartum using imputed data.

	T2 N = 324				T3 N = 324			
	b	CI	*p*	d	b	CI	*p*	d
SWEMWBS well-being	0.12	[−0.77, 1.01]	0.79	0.03	−0.60	[−1.63, 0.44]	0.26	−0.15
BaM-13 Being a mother ¤	−0.02	[−1.35, 1.32]	0.98	−0.00				
Mother health	−0.22	[−0.61, 0.18]	0.29	−0.12	0.05	[−0.47, 0.58]	0.84	0.03
Mother life satisfaction	−0.21	[−0.63, 0.22]	0.35	−0.12	−0.28	[−0.74, 0.18]	0.23	−0.14
Loneliness ¤	−0.01	[−0.23, 0.21]	0.94	−0.01	0.17	[−0.11, 0.45]	0.23	0.17
Access to practical help	0.10	[−0.17, 0.35]	0.47	0.09	−0.02	[−0.31, 0.27]	0.89	−0.02
Access to somebody to talk to	0.11	[−0.05, 0.28]	0.17	0.17	0.03	[−0.18, 0.24]	0.77	0.04
Worries: Housing ¤	−0.38	[−0.85, 0.09]	0.11	−0.19	0.56	[0.06, 1.06]	0.03	0.30
Worries: Employment ¤	−0.26	[−0.74, 0.22]	0.29	−0.13	0.08	[−0.51, 0.68]	0.78	0.04
Worries: Relationship ¤	−0.14	[−0.59, 0.32]	0.56	−0.07	0.07	[−0.52, 0.66]	0.82	0.03
EPDS Postnatal depression ¤	0.33	[−0.78, 1.44]	0.56	0.07	−0.38	[−1.70, 0.94]	0.57	−0.07
Smoking regularly ¤	0.00	[−0.08, 0.09]	0.96	0.01	−0.02	[−0.13, 0.10]	0.77	−0.04
Smoking in house ¤	−0.03	[−0.06, −0.00]	0.05	−0.26	−0.03	[−0.09, 0.02]	0.24	−0.18
Started to breastfeed	−0.03	[−0.09, 0.04]	0.40	−0.10				
Still breastfeeding	0.06	[−0.05, 0.17]	0.31	0.12	0.04	[−0.07, 0.16]	0.46	0.09
Employed					−0.02	[−0.15, 0.10]	0.73	−0.04
PSS PS ¤					−0.09	[−0.68, 0.50]	0.76	−0.04
PSS LPS ¤					0.00	[−0.11, 0.11]	0.98	0.00
PRFQ PM ¤					0.05	[−0.06, 0.16]	0.37	0.13
PRFQ CMS					−0.03	[−0.15, 0.09]	0.65	−0.06
PRFQ IC					0.74	[−0.80, 2.29]	0.34	0.12

Notes: T2: 3 months postpartum, T3: 12 months postpartum, b: regression coefficient, CI: 95% confidence interval, *p*: *p*-value, d: Cohen’s d effect size, ¤: low score indicates better outcomes. T2: 284 observed + 40 imputed. T3: 248 observed + 76 imputed.

## Data Availability

The datasets generated and analyzed during the current study are not publicly available to protect participant privacy but are available from the corresponding author upon reasonable request. Trial registration: The study was registered on 6 September 2018, at Clinicaltrials.gov NCT03659721. https://clinicaltrials.gov/ct2/show/NCT03659721 (accessed on 2 April 2025).

## Supplementary Materials

The following supporting information can be downloaded at: https://www.mdpi.com/article/10.3390/healthcare13131505/s1, Table S1: Comparison of baseline characteristics between families with low concern and families with medium or high concern; Table S2: Interaction analyses with regression coefficients and *p*-values. Table S3: Sensitivity analyses.

## Abbreviations

The following abbreviations are used in this manuscript:

FACAM	Family Clinic and Municipality
CAU	Care-as-Usual
CONSORT	Consolidated Standards of Reporting Trials
RCT	Randomized Controlled Trial
ADBB	Alarm Distress Baby Scale
COS-P	Circle of Security Parenting
P-PRFQ	Prenatal Parental Reflective Functioning Questionnaire
PRFQ	Parental Reflective Functioning Questionnaire
SWEMWBS	Short Warwick-Edinburgh Mental Wellbeing Scale
HADS	Hospital Anxiety and Depression Scale
ECR-S	Experiences in Close Relationship Scale-Short Form
PTSD-8	Post-Traumatic Stress Disorder Scale
ACE-10	Adverse Childhood Experience Questionnaire
EPDS	Edinburgh Postnatal Depression Scale
BaM-13	Being a Mother Scale
PSS	Parenting Stress Scale
IV	Instrumental Variable
SD	Standard Deviation
CI	Confidence Interval
