# Supplementary material for: Effects on Maternal Mental Health and Parental Functioning of an Interdisciplinary Intervention to Support Women in Vulnerable Positions Through Pregnancy and Early Motherhood: A Randomized Controlled Trial
Source: Healthcare (Basel). 2025 Jun 24;13(13):1505. doi: 10.3390/healthcare13131505 (PMC12248663; doi:10.3390/healthcare13131505)
Supplement: Supplementary file 1 [file healthcare-13-01505-s001.zip › healthcare-3598103-supplementary.docx]

Supplementary Table S1. Comparison of baseline characteristics at baseline between families with low concern and families with medium or high concern.

|  | Type | Low concern  N = 240 | | Medium or high concern  N = 91 | | Difference | |
| --- | --- | --- | --- | --- | --- | --- | --- |
|  |  | M/P | SD | M/P | SD | T-stat | p-value |
| Mother age (year) | M | 30.48 | (5.32) | 27.42 | (5.50) | 4.64 | **0.00** |
| Mother health | M | 7.00 | (1.81) | 7.48 | (1.72) | -2.18 | **0.03** |
| Mother life satisfaction | M | 7.71 | (1.83) | 7.79 | (1.95) | -0.36 | 0.72 |
| SWEMWBS well-being | M | 23.54 | (3.89) | 24.33 | (4.51) | -1.58 | 0.11 |
| Loneliness ¤ | M | 2.48 | (0.92) | 2.54 | (0.95) | -0.52 | 0.60 |
| Access to practical help | M | 3.99 | (1.05) | 4.21 | (0.98) | -1.74 | 0.08 |
| Access to somebody to talk to | M | 4.54 | (0.83) | 4.53 | (0.85) | 0.14 | 0.89 |
| HADS anxiety ¤ | M | 6.89 | (3.75) | 6.52 | (4.11) | 0.78 | 0.43 |
| HADS depression ¤ | M | 4.59 | (3.36) | 3.89 | (3.03) | 1.73 | 0.08 |
| PTSD total score ¤ | M | 13.62 | (5.86) | 14.71 | (6.25) | -1.49 | 0.14 |
| ECR fear of abandonment ¤ | M | 18.95 | (7.12) | 19.05 | (6.85) | -0.12 | 0.90 |
| ECR fear of intimacy ¤ | M | 13.50 | (6.55) | 13.84 | (7.11) | -0.40 | 0.69 |
| P-PRFQ opacity of mental states | M | 4.62 | (1.23) | 4.51 | (1.36) | 0.68 | 0.50 |
| P-PRFQ reflecting on the fetus-child | M | 4.92 | (1.05) | 5.40 | (0.86) | -3.76 | **0.00** |
| P-PRFQ the dynamic nature of mental states | M | 4.46 | (1.08) | 4.44 | (1.14) | 0.14 | 0.89 |
| ACE total score ¤ | M | 2.23 | (2.10) | 2.98 | (2.48) | -2.63 | **0.01** |
| Units of alcohol prior to pregnancy | M | 1.52 | (1.97) | 1.46 | (2.50) | 0.24 | 0.81 |
| Units of alcohol during pregnancy | M | 0.03 | (0.18) | 0.00 | (0.00) | 1.31 | 0.19 |
| Expecting first child | M | 0.60 | (0.49) | 0.69 | (0.46) | -1.62 | 0.11 |
| Cohabit with partner | P | 0.86 | (0.35) | 0.71 | (0.45) | 3.07 | **0.00** |
| Only speak Danish at home | P | 0.83 | (0.38) | 0.80 | (0.40) | 0.57 | 0.57 |
| High school or less | P | 0.31 | (0.46) | 0.69 | (0.46) | -6.73 | **0.00** |
| Vocational or secondary education | P | 0.23 | (0.42) | 0.16 | (0.37) | 1.20 | 0.23 |
| College, Bachelor, tertiary, or longer education | P | 0.47 | (0.50) | 0.14 | (0.35) | 5.67 | **0.00** |
| Employed | P | 0.42 | (0.49) | 0.21 | (0.41) | 3.58 | **0.00** |
| Sick leave | P | 0.20 | (0.40) | 0.10 | (0.30) | 2.11 | **0.04** |
| Unemployment benefit | P | 0.07 | (0.26) | 0.04 | (0.21) | 0.89 | 0.37 |
| Social assistance/unemployment program | P | 0.08 | (0.27) | 0.40 | (0.49) | -7.44 | **0.00** |
| In education | P | 0.19 | (0.39) | 0.19 | (0.39) | 0.01 | 0.99 |
| Unemployment no benefits | P | 0.02 | (0.13) | 0.02 | (0.15) | -0.32 | 0.75 |
| Smoking regularly | P | 0.08 | (0.28) | 0.23 | (0.42) | -3.70 | **0.00** |
| Never regularly used drugs like hash, pot, marihuana | P | 0.93 | (0.26) | 0.75 | (0.44) | 4.50 | **0.00** |
| Never regularly used drugs like amphetamine, ecstasy, cocaine, LSD | P | 0.96 | (0.19) | 0.90 | (0.30) | 2.21 | **0.03** |
| Used medicine during pregnancy (including non-prescription painkillers) | P | 0.63 | (0.48) | 0.54 | (0.50) | 1.58 | 0.12 |
| Expect to breastfeed | P | 0.97 | (0.17) | 0.95 | (0.22) | 0.72 | 0.48 |

Notes: M: Mean, P: Proportion, SD: Standard Deviation, T-Stat: T statistic, p: p-value, ¤: low score is best, Bold is significant at p<0.05.

Supplementary Table S2. Interaction analyses with regression coefficients and p-values based on imputed data.

|  | Additional effect of mother having high school education or less | | Additional effect of mother having high level of concern | | Additional effect of mother having high trauma level | |
| --- | --- | --- | --- | --- | --- | --- |
|  | b | p | b | p | b | p |
| SWEMWBS well-being | -0.44 | 0.67 | -0.34 | 0.77 | 0.15 | 0.87 |
| Mother health | 0.72 | 0.22 | -0.96 | 0.11 | -0.58 | 0.26 |
| Mother life satisfaction | -0.99 | 0.07 | -0.66 | 0.22 | -0.55 | 0.30 |
| Loneliness ¤ | 0.13 | 0.60 | 0.10 | 0.69 | 0.24 | 0.30 |
| Access to practical help | -0.13 | 0.65 | 0.09 | 0.78 | -0.20 | 0.49 |
| Access to somebody to talk to | -0.17 | 0.41 | 0.14 | 0.61 | -0.26 | 0.20 |
| Worries: Housing ¤ | -0.02 | 0.96 | -0.46 | 0.45 | 0.36 | 0.47 |
| Worries: Employment ¤ | 0.05 | 0.93 | -0.01 | 0.98 | -0.61 | 0.27 |
| Worries: Relationship ¤ | 0.19 | 0.72 | -0.17 | 0.79 | -0.26 | 0.61 |
| EPDS Postnatal depression ¤ | 1.42 | 0.29 | 0.70 | 0.66 | 1.26 | 0.33 |
| Smoking regularly ¤ | 0.01 | 0.95 | -0.04 | 0.78 | 0.01 | 0.93 |
| Smoking in house ¤ | -0.12 | 0.06 | -0.12 | 0.15 | 0.04 | 0.43 |
| Still breastfeeding | -0.02 | 0.87 | -0.01 | 0.96 | 0.17 | 0.15 |
| Employed | -0.03 | 0.76 | -0.05 | 0.73 | -0.08 | 0.47 |
| PSS PS ¤ | 0.35 | 0.82 | 0.03 | 0.99 | 0.26 | 0.86 |
| PSS LPS ¤ | -0.60 | 0.47 | 0.55 | 0.53 | -1.19 | 0.11 |
| PRFQ PM ¤ | -0.45 | 0.68 | -1.28 | 0.32 | 1.54 | 0.11 |
| PRFQ CMS | -0.47 | 0.77 | 0.54 | 0.75 | -2.42 | 0.12 |
| PRFQ IC | 2.01 | 0.11 | -0.87 | 0.49 | 0.30 | 0.79 |

Notes: N = 324, b: regression estimate, p: p-value, ¤: low score is best.

Supplementary table S3. Sensitivity analyses of maternal outcomes using complete case and instrumental variable analysis at 3 and 12 months postpartum.

|  |  | Complete case | | | |  | Instrumental variable analysis | | | |
| --- | --- | --- | --- | --- | --- | --- | --- | --- | --- | --- |
|  | T2 | |  | T3 | |  | T2 | | T3 | |
|  | b | p | d | b | p | d | b | p | b | p |
| SWEMWBS Well-being | 0.21 | 0.64 | 0.05 | -0.54 | 0.26 | -0.14 | 0.23 | 0.64 | -0.60 | 0.25 |
| BAM-13 Being a Mother ¤ | -0.11 | 0.87 | -0.02 |  |  |  | -0.12 | 0.87 |  |  |
| Mother health | -0.12 | 0.55 | -0.07 | 0.14 | 0.59 | 0.07 | -0.14 | 0.55 | 0.16 | 0.59 |
| Mother life satisfaction | -0.20 | 0.36 | -0.11 | -0.14 | 0.58 | -0.07 | -0.22 | 0.36 | -0.16 | 0.58 |
| Loneliness ¤ | -0.02 | 0.85 | -0.02 | 0.20 | 0.12 | 0.20 | -0.02 | 0.85 | 0.22 | 0.12 |
| Access to practical help | 0.05 | 0.70 | 0.05 | -0.09 | 0.54 | -0.08 | 0.06 | 0.70 | -0.10 | 0.53 |
| Access to somebody to talk to | 0.09 | 0.28 | 0.13 | -0.06 | 0.58 | -0.07 | 0.10 | 0.28 | -0.06 | 0.58 |
| Worries: Housing ¤ | -0.30 | 0.21 | -0.15 | 0.46 | 0.05 | 0.25 | -0.33 | 0.21 | 0.52 | **0.04** |
| Worries: Employment ¤ | -0.18 | 0.46 | -0.09 | 0.12 | 0.65 | 0.06 | -0.20 | 0.45 | 0.14 | 0.65 |
| Worries: Relationship ¤ | -0.15 | 0.52 | -0.08 | 0.11 | 0.68 | 0.05 | -0.17 | 0.52 | 0.12 | 0.67 |
| EPDS Postnatal depression ¤ | 0.12 | 0.83 | 0.02 | -0.14 | 0.83 | -0.03 | 0.13 | 0.83 | -0.16 | 0.82 |
| Smoking regularly¤ | -0.01 | 0.84 | -0.02 | -0.01 | 0.90 | -0.02 | -0.01 | 0.84 | -0.01 | 0.90 |
| Smoking in house ¤ | -0.03 | 0.051 | -0.26 | -0.01 | 0.48 | -0.09 | -0.03 | **0.04** | -0.01 | 0.48 |
| Started to breastfeed | -0.02 | 0.43 | -0.09 |  |  |  | -0.03 | 0.42 |  |  |
| Still breastfeeding | 0.06 | 0.29 | 0.12 | 0.05 | 0.45 | 0.10 | 0.07 | 0.29 | 0.05 | 0.44 |
| Employed |  |  |  | -0.05 | 0.47 | -0.09 |  |  | -0.05 | 0.47 |
| PSS PS ¤ |  |  |  | 0.86 | 0.25 | 0.15 |  |  | 0.96 | 0.25 |
| PSS LPS ¤ |  |  |  | 0.06 | 0.88 | 0.02 |  |  | 0.06 | 0.88 |
| PRFQ PM ¤ |  |  |  | -0.55 | 0.26 | -0.15 |  |  | -0.62 | 0.25 |
| PRFQ CMS |  |  |  | -1.44 | 0.07 | -0.23 |  |  | -1.62 | 0.07 |
| PRFQ IC |  |  |  | -0.16 | 0.78 | -0.04 |  |  | -0.19 | 0.77 |
| Observations | N = 284 | |  | N = 248 | |  | N = 284 | | N = 248 | |

Notes: T2: 3 months postpartum, T3: 12 months postpartum, b: regression estimate, p: p value, d: Cohens d effect size, ¤:low score is best, Bold is significant at p<0.05.
